# Model Systems for Evidencing the Mediator Role of Riboflavin in the UVA Cross-Linking Treatment of Keratoconus

**DOI:** 10.3390/molecules27010190

**Published:** 2021-12-29

**Authors:** Mihaela Monica Constantin, Cătălina Gabriela Corbu, Sorin Mocanu, Elena Irina Popescu, Marin Micutz, Teodora Staicu, Raluca Şomoghi, Bogdan Trică, Vlad Tudor Popa, Aurica Precupas, Iulia Matei, Gabriela Ionita

**Affiliations:** 1 Oftaclinic Clinic, Bd. Marasesti 2B, 040254 Bucharest, Romania; mihaelamonicaconstantin@yahoo.com (M.M.C.); carol_corbu@yahoo.com (C.G.C.); 2 Clinical Hospital of Ophthalmologic Emergencies, Alexandru Lahovari 1 Square, 010464 Bucharest, Romania; 3 “Ilie Murgulescu” Institute of Physical Chemistry of the Romanian Academy, Splaiul Independentei 202, 060021 Bucharest, Romania; smocanu@icf.ro (S.M.); elenairinapopescu@gmail.com (E.I.P.); vtpopa@icf.ro (V.T.P.); iulia.matei@yahoo.com (I.M.); 4 Department of Physical Chemistry, Faculty of Chemistry, University of Bucharest, Bd. Regina Elisabeta 4-12, 030018 Bucharest, Romania; teos@gw-chimie.math.unibuc.ro; 5 Chemistry Department, Faculty of Petroleum Technology and Petrochemistry, Petroleum-Gas University of Ploiesti, Bd. Bucuresti 39, 100680 Ploiesti, Romania; r.somoghi@gmail.com; 6 Department of Bioresources, National Institute for Research & Development in Chemistry and Petrochemistry—ICECHIM, Splaiul Independentei nr. 202, Sector 6, 060021 Bucharest, Romania; trica.bogdan@gmail.com

**Keywords:** collagen, riboflavin, hyaluronic acid, EPR spectroscopy, keratoconus, STEM

## Abstract

Riboflavin under UVA radiation generates reactive oxygen species (ROS) that can induce various changes in biological systems. Under controlled conditions, these processes can be used in some treatments for ocular or dermal diseases. For instance, corneal cross-linking (CXL) treatment of keratoconus involves UVA irradiation combined with riboflavin aiming to induce the formation of new collagen fibrils in cornea. To reduce the damaging effect of ROS formed in the presence of riboflavin and UVA, the CXL treatment is performed with the addition of polysaccharides (dextran). Hyaluronic acid is a polysaccharide that can be found in the aqueous layer of the tear film. In many cases, keratoconus patients also present dry eye syndrome that can be reduced by the application of topical solutions containing hyaluronic acid. This study presents physico-chemical evidence on the effect of riboflavin on collagen fibril formation revealed by the following methods: differential scanning microcalorimetry, rheology, and STEM images. The collagen used was extracted from calf skin that contains type I collagen similar to that found in the eye. Spin trapping experiments on collagen/hyaluronic acid/riboflavin solutions evidenced the formation of ROS species by electron paramagnetic resonance measurements.

## 1. Introduction

Keratoconus (KC) is an ocular disease with a relatively high prevalence (approximately 1:2000) [1] usually diagnosed during the second or third decade of life that causes an irregularly shaped cornea leading to severe impairment of vision. This disease is characterized by progressive thinning of the cornea, giving rise to a cone-shaped cornea instead of the normal spherical shape [1,2]. Hence, understanding the pathogenesis of this disease and controlling its evolution have attracted the attention of specialists in ophthalmology, and their efforts were accompanied by those of researchers in other fields, such as biochemistry, physics, or chemistry. Dry eye is also a symptom and a disease accompanying KC, with some studies including it in the multifactorial etiology of KC [3].

A relatively new technique introduced in 2003 by Wollensak et al. [4] is the corneal collagen cross-linking (CXL), based on the combined use of the photosensitizer riboflavin and UVA light of 370 nm. This method exploits the property of riboflavin to generate, under UVA irradiation, reactive oxygen species that further interact with biological assemblies [5]. The CXL method is also applied for the treatment of other diseases associated with the extracellular matrix involving various veins [6], degraded dentine [7], or the eye (pellucid marginal corneal degeneration, post-LASIK ectasia, infectious keratitis, bullous keratopathy) [8,9,10,11]. Although numerous clinical trials involving patients with KC are dedicated to the analysis of the efficacy and effects of CXL, literature data are scarce on the physicochemical aspects behind this treatment. In a recent study, we investigated the changes in the protein profile in tears collected from patients with dry eye syndrome and KC during treatment and explored the possible correlation of the electron paramagnetic resonance (EPR) parameters of spin probes that bind to some tear proteins with the composition of tears or ophthalmic parameters [12]. Lactoferrin (LF), lysozyme (LYZ) and lipocalin (a prealbumin protein) are among the proteins most abundant in tears [13,14,15]. The level of human serum albumin (HSA) may increase in tear secretions of patients suffering from dry eye syndrome and KC [16]. With this study, we aimed to find evidence of changes in collagen properties induced by riboflavin in the presence of UVA, and to identify the type of reactive species formed in various systems relevant for the CXL treatment of KC. Fibrous collagenous structures (type I, II and III) are present in the connective tissues of the eye, such as the cornea, sclera, vitreous body, retina, and also in other tissues like the skin, bones, and tendons [17]. To get information on how riboflavin influences the properties of collagen and the process of fibrillogenesis, collagen extracted from calf skin was used. This extract has type I collagen as the main component [18]. Also, we analyzed the influence of hyaluronic acid on fibrillogenesis. The present study involved rheological, microcalorimetric, electron microscopy, and electron paramagnetic resonance measurements. The rheological data evidenced the mechanical properties of the systems, while the microcalorimetric measurements provided information on the thermodynamic stability of collagen and collagen fibrils. Using three spin trapping agents, DMPO, CPH, and TEMP, EPR spectroscopy evidenced the reactive radical species formed in samples containing collagen, in solutions of proteins present in tears (LF, LYZ, HSA), and in tears collected from patients during CXL treatment.

## 2. Results and Discussion

The collagen systems investigated in this study are described in Table 1. These systems were characterized by rheology, STEM, and microDSC methods at a pH value of 3.5 or 7.5 in order to get bulk information and microscopic information on collagen systems.

**Figure 1:**
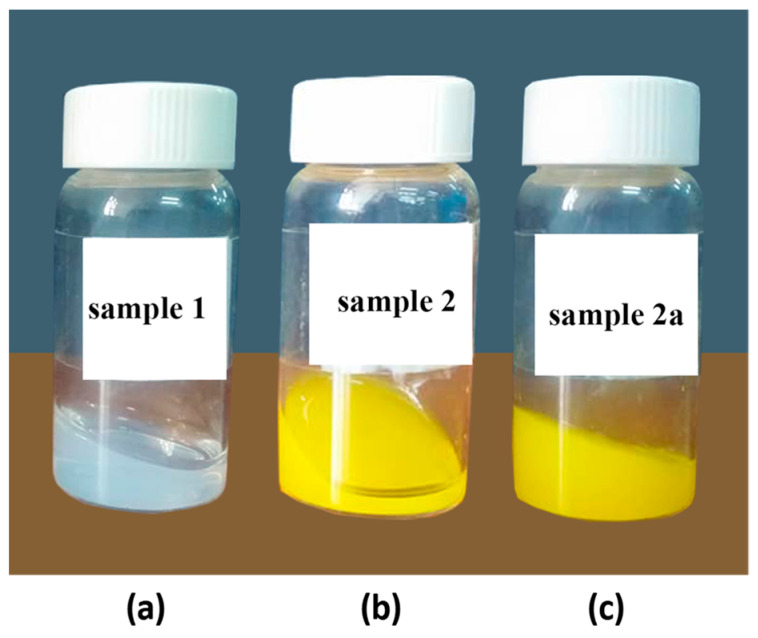
Collagen gels formed at pH 7.5 in the absence of riboflavin (**a**) and in the presence of riboflavin prior to (**b**) and after (**c**) UVA irradiation.

Fibril formation in collagen solutions was initiated following the procedure described in [16,17,18,19,20,21,22,23,24,25]. Fibril formation in all collagen systems under investigation occurs only at pH 7.5. For instance, Figure 1 presents the gels formed in the absence of riboflavin (Figure 1a), in the presence of riboflavin (Figure 1b), and by the irradiated collagen/riboflavin sample (Figure 1c).

The spin trapping experiments were performed for samples **1a**–**4a**, for solutions of proteins that can be found in tears, for a pharmaceutic solution containing riboflavin and dextran (Peschke solution) used in CLX treatment and for tears collected from patients during the CLX treatment.

### 2.1. Rheological Measurements

**Figure 2:**
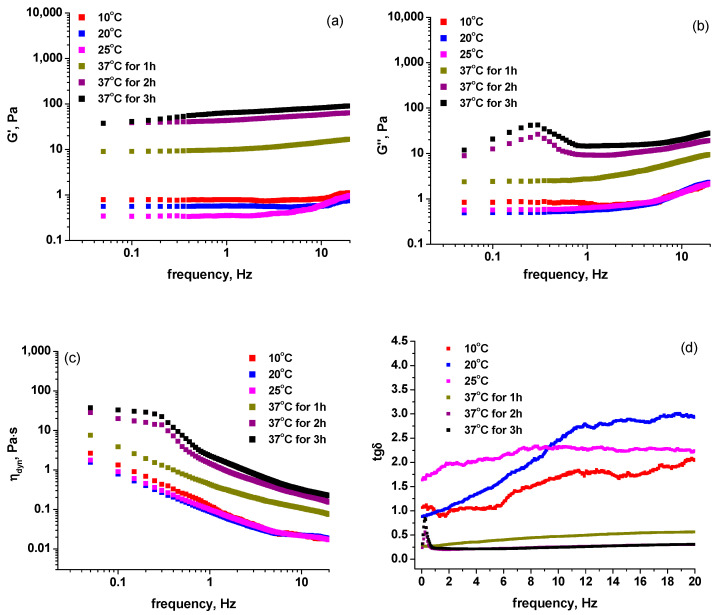

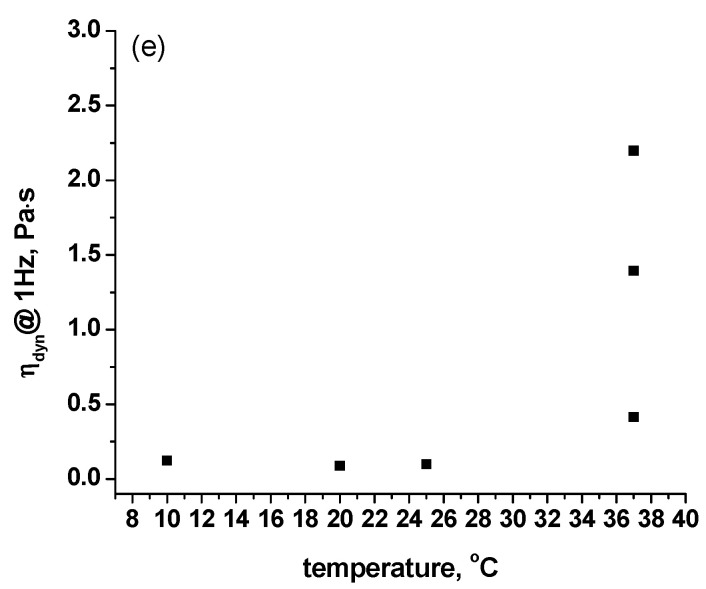
The variation of (**a**) storage modulus (G’), (**b**) loss modulus (G”), (**c**) dynamic viscosity (η_dyn_ = G”/ω) and (**d**) loss tangent (tgδ = G”/G’) with frequency, and the variation of η_dyn_ with temperature (**e**) for sample **1**.

Rheological data for samples prepared at pH 7.5 were acquired at four temperatures in the range 10–37 °C: 10, 20, 25, and 37 °C. As the process of collagen fibril formation is favored at 37 °C, rheometric measurements at this temperature were performed at different times from the onset of gelation (1, 2 and 3 h). The rheological behavior of the samples described in Table 1 is similar. For instance, in the case of sample **1**, the variation of the rheological parameters (storage modulus G’, loss modulus G”, dynamic viscosity η_dyn_ = G”/ω and loss tangent tgδ = G”/G’, where ω is angular frequency in rad s^−1^) as a function of frequency (in Hz) is illustrated in Figure 2a–d, while the change in the dynamic viscosity corresponding to a frequency of 1 Hz as a function of temperature is shown in Figure 2e. The rheological behavior of samples **2**–**4** and **1a**–**4a** is presented in the supplementary material file (Figures S1–S7). In the temperature range of 10–25 °C, all samples behave as viscoelastic liquids, while at the temperature of 37 °C the rheological parameters correspond to a gel system as the fibril formation occurs. At 37 °C, G’ >> G” over the whole frequency domain, and the values of G’, G” and η_dyn_ are at least an order of magnitude higher than at lower temperatures, depending on the time the system has been maintained at the gelation temperature. At the gelation temperature, the loss tangent has sub-unitary values over the frequency domain. Figure 2e shows that the dynamic viscosity η_dyn_ corresponding to 1 Hz increases suddenly at the gelation temperature after incubation from 1 to 3 h.

The influence of riboflavin, hyaluronic acid and UVA treatment on fibril formation can be evidenced by changes in the G’ value, as indicated in Table 2 for an angular frequency of 1 Hz and the temperatures indicated above. In the case of sample **1a** corresponding to a collagen sample exposed to UVA radiation, it can be noticed that the steep increase in dynamic viscosity at 37 °C is more pronounced (Figure S1d). In the case of sample **2** containing riboflavin, higher values of G’, G” and η_dyn_ (at 37 °C, after 1 h and 2 h of equilibration) were noticed as compared to sample **1** (Figure S2a–d). This indicates that gelation occurs faster as it is favored by the presence of riboflavin.

The UVA irradiation of the collagen-riboflavin system (sample **2a**, Figure S3) determines a different rheological behavior by comparison to the non-irradiated system. Gelation during collagen fibril formation is slower, and the completeness of gel formation after 3 h of fibril formation led to a much weaker physical gel. For instance, the G’, G” and η_dyn_ values of sample **2a** are lower compared to those of sample **2** (see Figures S2 and S3). This behavior suggests that UVA exposure in sample **2a** impacted the processes occurring in the collagen-riboflavin system. Under UVA radiation, riboflavin undergoes photo degradation processes involving intramolecular photodealkylation and/or photo addition corresponding to the excited singlet state, or intramolecular photo reduction corresponding to the excited triplet state [26]. Free radical formation accompanying the photodegradation of riboflavin may initiate the intermolecular cross-linking of collagen prior to the onset of the collagen fibril formation process.

Samples **3** and **3a** are characterized by the formation of well-defined strong gels at 37 °C even for the case of 1 h equilibration time (Figures S4 and S5). This stands for a physical interaction between collagen and hyaluronic acid that adds to the fibril formation process. The rheograms almost overlap for the equilibration times of 2 h and 3 h at 37 °C (Figures S4a–c and S5a–c), which indicates that, in the presence of hyaluronic acid, the process of fibril formation is completed faster. This behavior is in good agreement with the favorable role played by hyaluronic acid in strengthening and improving the elasticity of the collagen gel resulting during fibrillogenesis [27]. Literature data showed that hyaluronic acid may experience photodegradation under the action of UV light that gradually affects its molecular weight, leading to the decrease of its intrinsic viscosity observed in saline solution (0.2 M NaCl) at 25 °C [28]. In the case of sample **3a**, the gels have a slightly lower consistency (Figure S5b–d) compared to the non-irradiated sample **3**.

Sample **4** reaches the gel state equilibrium in a shorter time, as dependences of G’ and G” as a function of frequency at 37 °C at different equilibration times are almost overlapped at applied frequencies higher than or equal to 1 Hz (Figure S6). The exposure of this tricomponent system to UVA light (sample **4a**) determines a different behavior compared to sample **4**. The behavior of sample **4a** is similar to that of sample **2a** (see Figures S3 and S7), and this result emphasizes the preponderant role played by riboflavin relative to hyaluronic acid in controlling the rheological properties of these mixed systems.

### 2.2. STEM Images

**Figure 3:**
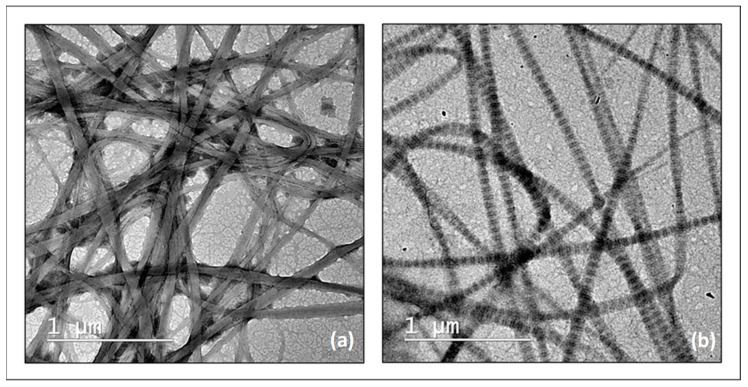
STEM images for collagen fibrils in the absence (**a**) and presence (**b**) of riboflavin, pH 7.5.

To evidence the role of riboflavin and hyaluronic acid on collagen fibrillogenesis, the samples have been examined after performing the negative staining procedure described in the experimental section. Figure 3 presents the STEM images obtained for sample **1** and sample **2**. The STEM images revealed a random formation of the fibrils in the case of sample **1**, which contains only collagen (Figure 3), while in the case of the collagen solutions containing riboflavin and/or hyaluronic acid, the fibrils display a clear axial and a regular transverse D-periodic banding pattern (Figure 3b). The distances between ridges are in the range of 65–70 nm, a value close to that of the native fibrillar structure of type I collagen [29,30]. This latter aspect proves the regulatory effect in collagen fibril formation displayed by both riboflavin and hyaluronic acid.

Rheological measurements and STEM investigation evidenced that both riboflavin and hyaluronic acid influence the fibrillogenesis of collagen. We further investigated by microDSC measurements the effect of riboflavin, hyaluronic acid, and UVA exposure of collagen samples on the thermal stability of the protein assemblies.

### 2.3. MicroDSC Measurements

In collagen samples, an increase in temperature determines the breaking of hydrogen bonds and induces irreversible unfolding of the triple helix to random coils [31]. The DSC signal obtained for collagen thermal denaturation displays one endothermic peak that can be decomposed into components that correspond to pre-, post-, and major transition. Literature data report the presence in the collagen DSC curve of either a main peak, attributed to triple helix melting, with a shoulder at lower temperature associated with the fibrillation of collagen [32], or one main peak located between two shoulders [33]. The number of transition peaks assigned to fibril denaturation varies from one peak to three peaks [34,35], the high-temperature peak corresponding to the melting of the fibrillar form of collagen, while the low temperature transition is related to the melting of monomeric collagen [36].

**Figure 4:**
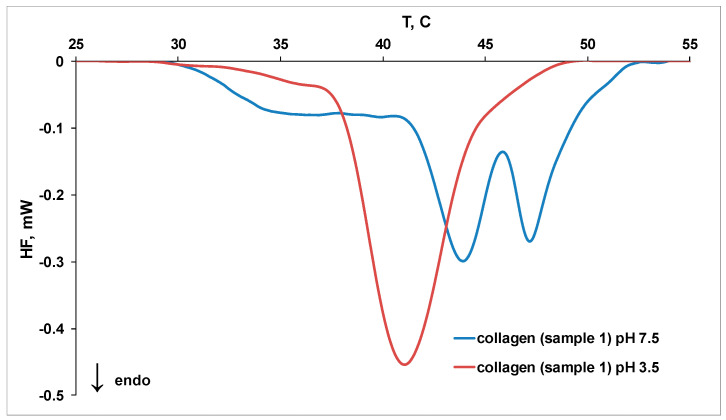
The DSC thermograms for thermal denaturation of sample **1** at pH 3.5 and 7.5.

The DSC thermograms for collagen thermal denaturation in different systems are discussed in the following. Figure 4 displays the influence of pH on collagen thermal denaturation and fibril formation. The DSC signal of sample 1 (in solution at pH 3.5 or 7.5) can be decomposed in three peaks (Figure S8). The major peak is attributed to the melting of the triple helix, while the minor peaks correspond to fibril denaturation [33].

Higher values of peak temperatures for collagen thermal denaturation (Table 3) were obtained at pH 7.5 and suggest a higher thermal stability of triple helix (T_peak2_) and fibrillar collagen (T_peak1_ and T_peak3_).

**Figure 5:**
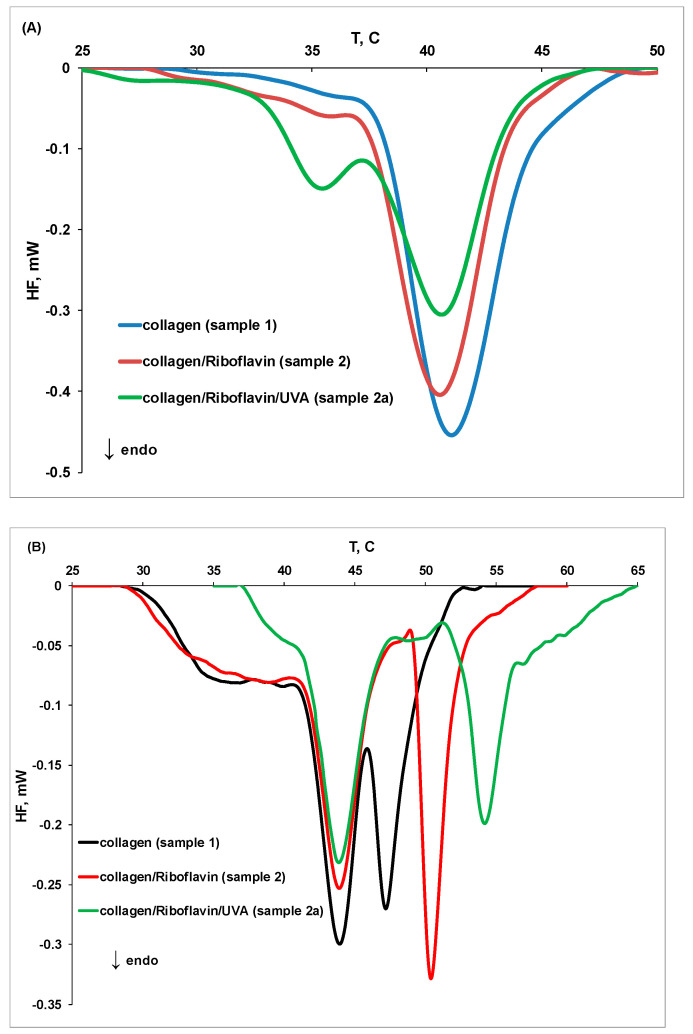
The DSC thermograms for thermal denaturation of collagen (sample **1**), collagen in the presence of riboflavin (sample **2**), and collagen in the presence of riboflavin after UVA exposure (sample **2a**) at (**A**) pH 3.5 and (**B**) pH 7.5.

The influence of riboflavin and UVA irradiation on collagen thermal denaturation and fibril formation at different pH values is displayed in Figure 5. At pH 3.5, the collagen thermal stability is reduced in the presence of riboflavin (sample **2**) and in the presence of riboflavin and UVA irradiation (sample **2a**), as the peaks are shifted to lower values. An opposite effect was obtained at pH 7.5: riboflavin induces the formation of new collagen fibrils that show a better thermal stability (T_peak1_ and T_peak3_ increase), while the thermal stability of the triple helix structure remains unchanged. 

The exposure of collagen/riboflavin solution to UVA irradiation for 30 min (sample **2a**) shifts the first and third peaks to higher temperature values, indicating a higher thermal stability of collagen fibrils. Furthermore, an additional peak at higher temperature (T_peak4_) was observed, suggesting the formation of new collagen fibrils. Partial defibrillation of collagen under riboflavin/UVA radiation action might be an initial step that enables the formation of radicals on the protein chain, which further ensures a packing of the fibrils into a stronger network.

**Figure 6:**
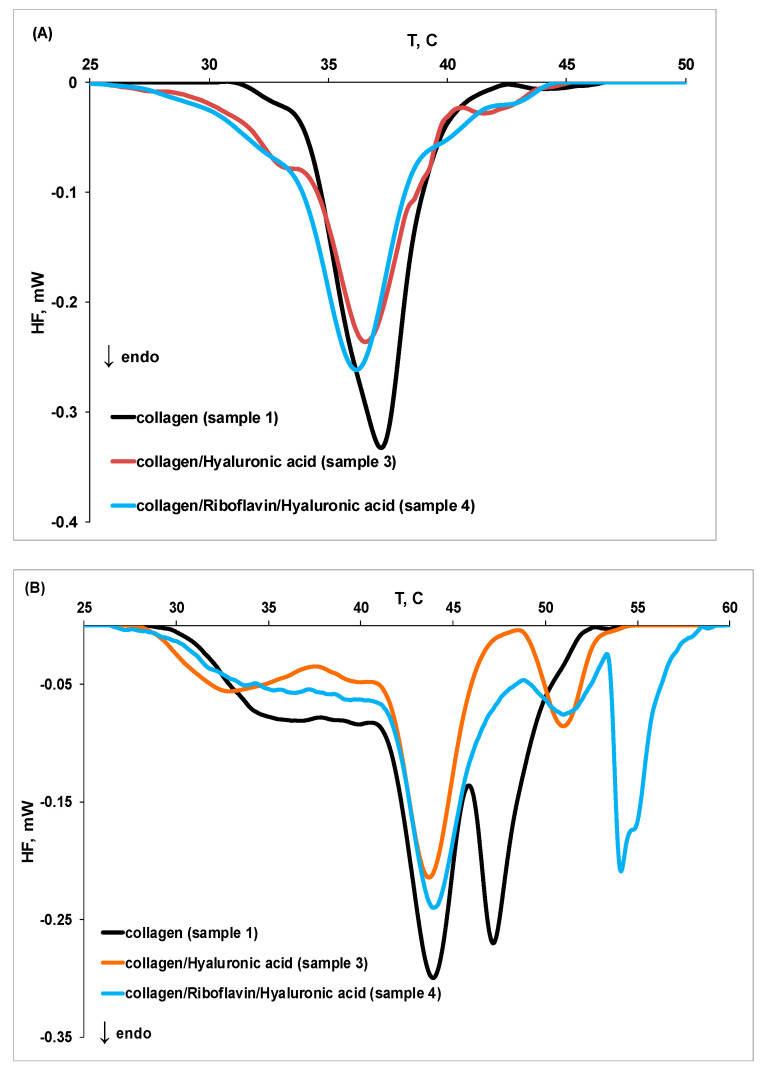
The DSC thermograms for thermal denaturation of collagen (sample **1**), collagen in the presence of hyaluronic acid (sample **3**), and collagen in the presence of hyaluronic acid and riboflavin (sample **4**) at (**A**) pH 3.5 and (**B**) pH 7.5.

Figure 6 presents the influence of hyaluronic acid and riboflavin (samples **3** and **4**) on collagen thermal stability at different pH values. At pH 3.5, the presence of hyaluronic acid and riboflavin (sample **4**) shifts the DSC peaks towards lower temperatures, indicating that these compounds favor defibrillation and denaturation of collagen. At pH 7.5, the destabilization effect induced by hyaluronic acid on collagen is reduced in the presence of riboflavin. Moreover, a fourth peak could be noticed at higher temperatures, suggesting that additional intermolecular cross-linking of collagen fibers occurs. Cross-linking stabilizes the fibrous structure of collagen by reducing the separation of the individual molecules [37]. Riboflavin also protects the new fibrillar collagen formed at higher temperature, as evidenced by the corresponding (higher value) denaturation peak (T_peak4_).

**Figure 7:**
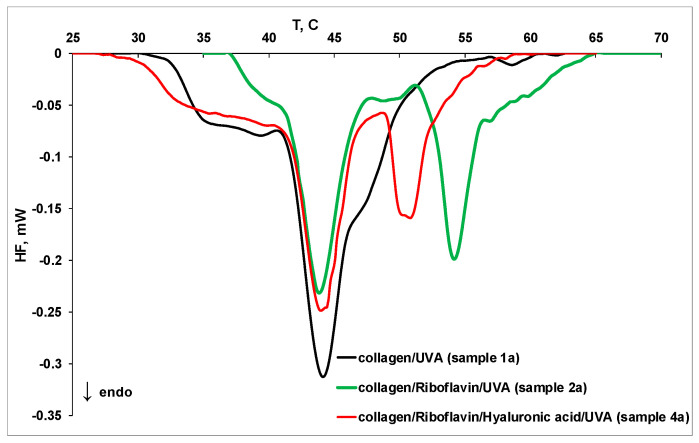
The DSC thermograms for thermal denaturation of collagen (sample **1a**), collagen/riboflavin (sample **2a**), and collagen/riboflavin/hyaluronic acid (sample **4a**) under UVA irradiation at pH 7.5.

Figure 7 displays the effect of UVA irradiation on collagen thermal denaturation in different systems at pH 7.5, corresponding to samples **1a**, **2a,** and **4a**. The exposure of collagen/riboflavin solution to UVA irradiation (sample **2a**) for 30 min shifts the peaks to higher temperatures and induces the presence of a fourth peak, suggesting the formation of new collagen fibrils with better thermal stability. Hyaluronic acid destabilizes the collagen, and the thermal stability of fibrils is reduced (sample **4a**). The influence of riboflavin, hyaluronic acid, and UVA irradiation on collagen thermal denaturation at pH 7.5 is summarized in Table 4.

Higher values of denaturation heat were obtained at pH 7.5 for all systems, indicating a higher content of fibrillar collagen. Riboflavin induces a stabilization effect on collagen fibrils, evidenced by the higher values of denaturation heat at both pH values. The exposure of collagen/riboflavin solution to UVA irradiation for 30 min decreases the denaturation heat at pH 7.5, indicating that a process of collagen partial defibrillation occurs. A significant destabilization effect on collagen is induced by hyaluronic acid, especially at pH 3.5.

**Figure 8:**
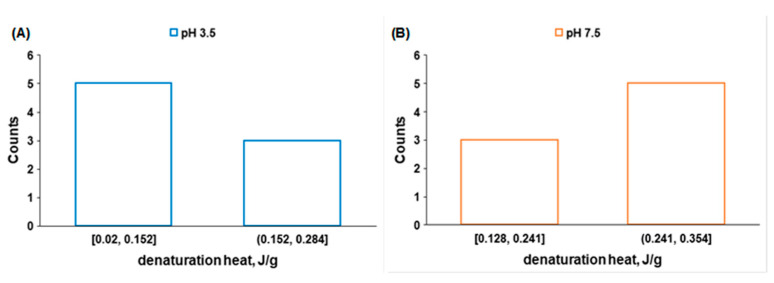
Denaturation heat histograms of collagen systems at pH 3.5 (**A**) and 7.5 (**B**).

The averages for the denaturation heat of collagen in different systems at pH 3.5 and 7.5 are 0.152 J/g and 0.241 J/g, respectively. These average values were used as references for the histograms presented in Figure 8. While at pH 7.5 there are 5 systems with denaturation heat above the average, at pH 3.5, the trend is opposite: only 3 systems have a higher-than-average denaturation heat. These results confirm the higher fibrillar content of collagen at pH 7.5.

The rheological and thermodynamic changes observed in the collagen systems exposed to riboflavin and UVA are the results of interactions between the reactive oxygen species (ROS) generated by the interaction of riboflavin with UVA light. In the next section, the information obtained by spin trapping experiments in various systems that contain riboflavin exposed to UVA and components of tear films (proteins, hyaluronic acid) are presented.

### 2.4. Radical Species Formed in Collagen/Riboflavin/UVA Systems

Under UVA light, riboflavin generates ROS, such as hydroxyl (HO^•^), superoxide (O_2_^•−^), hydroperoxide (HOO^•^) radicals, and singlet oxygen (^1^O_2_), following two mechanisms [38,39]. ROS generated by photodegradation of riboflavin mediate the formation of intra or interfibrillar bonds in collagen during CXL treatment, but their side effects cannot be omitted as free radicals may induce the death of endothelial cells, affecting the crystalline lens or retina. However, these effects are reduced by the presence in the composition of the tear film of proteins like lactoferrin (LF) and lysozyme (LYZ), which have the role of removing these free radicals. In addition, the Peschke solution used for CXL treatment contains dextran, which limits the concentration of oxygen-centered radicals generated by riboflavin and ensures a certain viscosity of the solution. Although the main process in CXL treatment regards the cross-linking of collagen, the interaction of the free radicals with components of the tear film also occurs. It is known that the keratoconus disease is often associated with dry eye syndrome (DES), which causes a low content of LF and LYZ in the tear film [3,15]. The treatment of DES involves different formulations for artificial tears that have hyaluronic acid as the main component, which can in fact be found in the mucin layer of the tear film.

By EPR measurements, we aim to highlight the short-lived free radicals (especially those centered on oxygen) formed in various systems exposed to riboflavin and UVA radiation. For this purpose, three spin traps have been used: 5,5-dimethyl-1-pyrroline N-oxide (DMPO), used to identify HO^•^ radicals, 1-hydroxy-3-carboxy-2,2,5,5-tetramethylpyrrolidine (CPH), used to identify O_2_^•−^ and HO^•^ radicals, and 2,2,6,6-tetramethylpiperidin-4-one (TEMP) used to identify ^1^O_2_ (Figure S9). CPH is a radical scavenger with higher sensitivity than DMPO in identifying HO^•^ and HOO^•^ [40]. The total protein content of the tear film is about 15 mg/mL, with LF and LYZ in concentrations up to 3 mg/mL. Human serum albumin (HSA) can be found in high concentration in tears only upon the rupture of blood vessels in the conjunctiva [15]. Therefore, the concentration of each protein in the solutions examined was 3 mg/mL. Determinations were also carried out in a mixture of the three proteins containing 0.1% hyaluronic acid.

To evidence the role of UVA irradiation, the EPR spectra of riboflavin solution were firstly recorded in the dark, noting the absence of a signal (the black spectrum in Figure S10). After exposure to UVA radiation, the 4-line EPR signal characteristic to the ^•^DMPO-OH adduct (hyperfine splitting constants a_N_ = 14.9 G and a_H_ = 14.7 G) is observed (cyan spectrum in Figure S10). The spin adducts were evidenced in the following systems: solution of LF, LYZ, and HSA in the presence of riboflavin 0.1%, mixture of the three proteins and hyaluronic acid, Peschke solution, sample **1a**, sample **4a**, and in tears collected from patients immediately after the CXL procedure.

**Figure 9:**
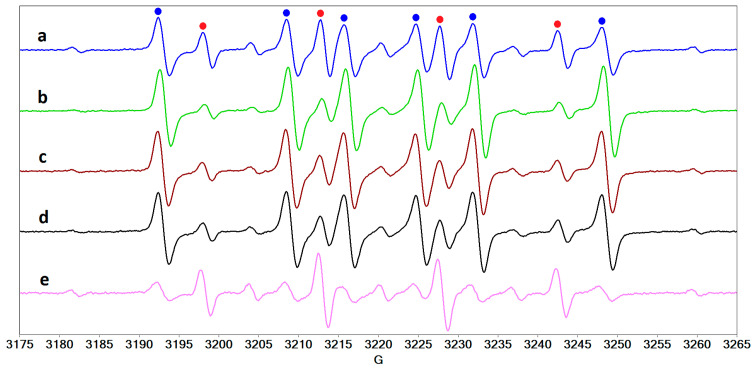
The EPR spectra of DMPO adducts formed after UVA exposure, in the presence of riboflavin, of a solution of (**a**) HSA, (**b**) LF, (**c**) LYZ, and of the mixture of the three proteins in the absence (**d**) and in the presence (**e**) of hyaluronic acid.

**Figure 10:**
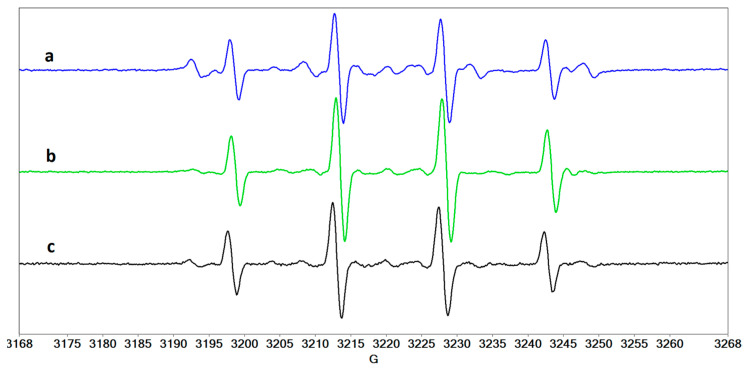
The EPR spectra of DMPO adducts formed after UVA exposure, in the presence of riboflavin, of collagen solution (**a**) in the absence of hyaluronic acid, (**b**) in the presence of hyaluronic acid, and (**c**) in the presence of the mixture of the three proteins and hyaluronic acid.

The EPR spectra of the spin adducts formed in the protein systems in the presence of riboflavin and UVA are a sum of two main components. One component corresponds to the ^•^DMPO-OH adduct, and the other to a DMPO adduct with a carbon-centered radical (hyperfine splitting constants a_N_ = 16.1 G and a_H_ = 23.3 G) formed probably by the interaction of oxygen-centered reactive radicals generated by riboflavin with the proteins. The nitroxide resulted by DMPO degradation is also evidenced but its contribution is less than 10% (see Table S1). The lines corresponding to each adduct are marked with a red dot for ^•^DMPO-OH and with a blue dot for the carbon-centered radical adduct (Figure 9). In the presence of hyaluronic acid or dextran, it was found that the formation of carbon-centered radicals is reduced. Thus, we can affirm that hyaluronic acid plays a role not only in restoring the integrity of the tear film, but also an antioxidant role against highly reactive radicals. We also observed the presence of HO^•^ and carbon-centered radicals in the solution containing collagen and riboflavin (sample **1a**) after exposure to UVA radiation (Figure 10a). The presence of hyaluronic acid (Figure 10b) and of the three proteins and hyaluronic acid (Figure 10c) reduces the contribution of the carbon-centered radical.

**Figure 11:**
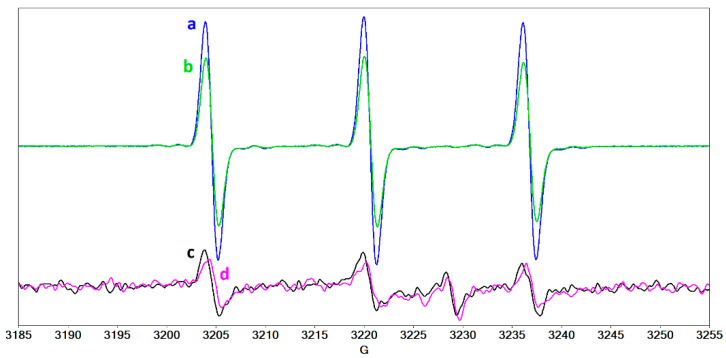
The EPR spectra of CPH adducts formed by UVA irradiation of a solution containing riboflavin and (**a**) collagen, (**b**) collagen/SOD, (**c**) collagen/hyaluronic acid, and (**d**) collagen/hyaluronic acid/SOD.

In addition to the HO^•^ radical, irradiation of riboflavin-containing solutions can lead to the formation of the O_2_^•−^ radical that, although having lower reactivity compared to the HO^•^ radical due to low-rate constant values [41], can be responsible for the generation of highly-reactive secondary species with high biological toxicity [42]. Such species may, in theory, have greater negative side effects in the treatment of CXL. The CPH spin trap can evidence the formation of this radical by use of superoxide dismutase (SOD). Figure 11 shows the EPR spectra of CPH adducts formed in collagen solution in the absence (Figure 11a) and in the presence (Figure 11b) of SOD. It can be observed that the intensity of the spectrum in the presence of SOD is slightly lower, which may lead to the conclusion that the O_2_^•−^ radical is generated in collagen solution. In the case of the solution containing collagen and hyaluronic acid, the intensities of the two spectra are similar and lower (Figure 11c,d). This result highlights the protective role that hyaluronic acid may play during CXL treatment. However, it should be underlined that we did not perform quantitative measurements to determine the concentration of the radical species formed; therefore the formation of the O_2_^•−^ species in this system is not certain.

**Figure 12:**
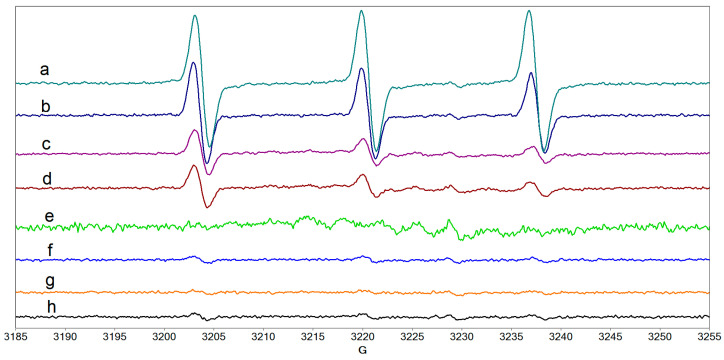
The EPR spectra of TEMPONE formed after UVA irradiation of the following systems: (**a**) water, (**b**) collagen/riboflavin, (**c**) collagen, (**d**) Peschke solution, (**e**) tears, (**f**) collagen/hyaluronic acid, (**g**) collagen/hyaluronic acid/HSA/LF, (**h**) HSA/LF/LYZ.

The next step was to examine the possible formation of ^1^O_2_ that poses the highest risk of eye tissue destruction following CXL treatment. Several systems have been evaluated to ascertain the presence of this radical by generating TEMPONE (4-oxo-TEMPO). Figure 12 shows that TEMPONE can be generated in riboflavin solution, in collagen solution containing riboflavin, in collagen solution, but also in the Peschke solution containing riboflavin and dextran. In contrast, TEMPONE was not present in tears, in a mixture of collagen and hyaluronic acid, or in a mixture of collagen, hyaluronic acid, and protein. This set of determinations emphasizes, once again, the protective role of hyaluronic acid and of the proteins present in tears.

## 3. Materials and Methods

5,5-dimethyl-1-pyrroline-N-oxide (DMPO), 2,2,6,6-tetramethylpiperidine (TEMP), superoxide dismutase (SOD), lysozyme, lactoferrin, and human serum albumin were purchased from Sigma Aldrich. 1-hydroxy-3-carboxy-2,2,5,5-tetramethylpyrrolidine (CPH) was obtained from Enzo. Riboflavin and hyaluronic acid were purchased from Roth and Acros Organics, respectively. The Peschke D solution was obtained from Lightmed.

### 3.1. Collagen Extraction and Purification

Collagen type I was extracted from calf skin following a modified procedure adapted from refs. [43,44,45,46,47,48,49,50], which is described in detail in the supplementary material file.

### 3.2. Sample Preparation

Collagen samples for STEM visualization of fibril formation in vitro, in the absence or in the presence of riboflavin and hyaluronic acid, were prepared in accordance to the procedure described in [19,20,21,22,23,24,25], as follows: 5 mL of phosphate-buffered saline (PBS) buffer solution were added (under vigorous magnetic stirring on ice) into the same volume of acetic solution of collagen or of its acetic mixtures with the same initial collagen concentration of 0.5%. The final pH of the so-prepared systems was adjusted to 7.5 using a concentrated aqueous NaOH solution (40%). Similar samples containing riboflavin and/or hyaluronic acid in concentration of 0.1% were prepared. The composition of PBS consisted of 270 mM NaCl, 60 mM Na_2_HPO_4,_ and 60 mM N-[tris(hydroxymethyl)methyl]-2-aminoethansulfonic acid (TES) [19], with a final pH set to 8.50 by adding concentrated NaOH solution. The collagen fibrillogenesis was triggered by incubating the mixtures at 37 °C for 4 h. After that, the process of fibril formation was assessed visually when turbid and physical cross-linked gels resulted. The collagen samples were studied at pH 3.5 and pH 7.5 by rheometry and microDSC. For evidencing the reactive species formed in collagen during UVA irradiation, tear secretions from patients were collected during exposure to UVA, and solutions of collagen in the absence and in the presence of hyaluronic acid and/or riboflavin were prepared.

For EPR measurements, stock solutions of each spin trapping agent were prepared in a concentration of 50 mM. The concentration of lysozyme, lactoferrin, and human serum albumin was 3 mg/mL while the concentration of collagen was 0.25%. For samples containing riboflavin and/or hyaluronic acid, the concentration was 0.1%. The tear samples were collected from patients during CXL treatment and mixed immediately with an equal volume of spin trapping agent solution, then the sample was transferred to a capillary that was introduced in liquid nitrogen prior to EPR measurement.

### 3.3. Instruments

#### 3.3.1. STEM

To visualize the fibril structure of collagen, the grids were exposed to the electron beam of the instrument (TEM Tecnai^TM^ G2 F20 TWIN Cryo-TEM, FEI Company^TM^) at an accelerating voltage of 200 kV. TEM samples were prepared in accordance with adapted protocols applied to collagen fibrils [19,51,52,53]. Thus, an aliquot extracted from each of the individual turbid hydrogels resulted after collagen fibrillogenesis was dispersed into PBS of pH 7.5 to give a final fibril suspension cca. 20-fold diluted. Then, a droplet of diluted suspension was poured onto a copper grid (holey carbon coated copper grid) for 5 min. The excess of solution was carefully removed by means of a piece of filter paper placed at the grid edge. The fibrils adsorbed onto the carbon-coated grid were negatively stained with a droplet of aqueous phosphotungstic acid solution (2–3%, pH 7.4) for 1–2 min. Then, the treated copper grid was rinsed with 3-4 droplets of distilled water and eventually air dried.

#### 3.3.2. Rheometry

The viscoelastic behavior of collagen and collagen-based systems and gel formation during collagen fibrillogenesis were investigated by dynamic rheometry. To this end, an MFR 2100 instrument (GBC, Australia) was employed, as previously described [54,55,56]. The rheological measurements were carried out at the following temperature values: 10, 20, 25, and 37 °C. A small volume of initial viscoelastic sample was rapidly sandwiched between the two chilled (5 °C) parallel plates of the rheometer equipped with a home-made temperature control jacket connected to a circulating water bath Lauda E100. Prior to performing the measurements, every single sample was equilibrated at 10, 20, and 25 °C for 15 min, except for 37 °C where the equilibration times were 1, 2, and 3 h. The values of storage and loss moduli (G’, G”), dynamic viscosity (η_dyn_), and loss tangent (tgδ) were determined using the following instrument parameters: pseudorandom noise oscillation, gap between plates 300 μm, oscillation amplitude 0.04 μm, frequency domain 0.05–20.00 Hz, and 30 scans per rheogram. To avoid water evaporation from the sample during fibrillogenesis (over 3 h), a tiny amount of low-viscous polydimethylsiloxane (viscosity of 5 mPa·s at 25 °C) was used to seal the system to be measured.

#### 3.3.3. MicroDSC

Thermal denaturation of collagen at pH 3.5 and 7.5 was studied using differential scanning microcalorimetry (MicroDSC). The measurements were carried out with a SETARAM MicroDSC III calorimeter in the temperature range 15–85 °C at a 1 °C min^−1^ heating rate. Experimental DSC data were analyzed using the Calisto v.1077 software package; thus, the denaturation temperature (T_peak_) and the denaturation heat were obtained. PeakFit 4.12 software was used to decompose the endothermic peak corresponding to the collagen thermal denaturation.

#### 3.3.4. EPR Spectroscopy

The EPR spectra of spin adducts were recorded on a JEOL FA 100 spectrometer equipped with a cylindrical-type resonator TE011, with a frequency modulation of 100 kHz, microwave power of 0.998 mW, sweep time of 480 s, modulation amplitude of 1 G, time constant of 0.3 s, and a magnetic field scan range of 100 G. For the spin trapping experiments, the following settings were used: sweep field 150 G, frequency 100 kHz, gain 800, sweep time 240 s, time constant 0.1 s, modulation width 1 G, and microwave power 1 mW. The collagen samples were exposed to UVA radiation (370 nm) using an UV irradiation device directly in the spectrometer’s cavity, as follows. The UV light generated by a mercury arc lamp (500 W, LOT-Quantum Design, Darmstadt, Germany) was passed through an UV irradiation accessory (ES-UVAT1, JEOL Resonance Inc., Tokyo, Japan; 370 nm HOYA colored optical glass filter ES-13020FL) connected to the spectrometer cavity via a condenser lens (ES-UVLL/UVLS, JEOL Resonance Inc.). The time of irradiation was 30 min. The EPR spectral simulations of spin adducts were performed using the WinSim program [57,58,59].

## 4. Conclusions

In conclusion, we can state that the results of our physicochemical determinations on systems containing collagen and hyaluronic acid exposed to the action of riboflavin and UVA irradiation support the roles that riboflavin and hyaluronic acid play in the treatment of CXL. Riboflavin, by generating free radicals, ensures the formation of interfibrillary bonds that lead to an increased mechanical strength of the cornea, while hyaluronic acid has the role of regulating or neutralizing some of the free radicals formed. Dextran has similar role to that of hyaluronic acid.

## Figures and Tables

**Table 1:** The composition of collagen samples investigated in this study, in the absence and in the presence of UVA light.

Sample	Collagen 0.25%	Riboflavin 0.1%	Hyaluronic Acid 0.1%	UVA Exposure
**1**	x	-	-	-
**1a**	x	-	-	x
**2**	x	x	-	-
**2a**	x	x	-	x
**3**	x	-	x	-
**3a**	x	-	x	x
**4**	x	x	x	-
**4a**	x	x	x	x

**Table 2:** The G’ values (in Pa) for samples **1**–**4** and **1a**–**4a** corresponding to a frequency value of 1 Hz.

Sample	10 °C	20 °C	25 °C	37 °C (1 h)	37 °C (2 h)	37 °C (3 h)
**1**	0.79	0.57	0.36	9.90	43.43	63.88
**1a**	0.66	0.66	0.53	14.16	77.04	149.10
**2**	0.77	0.49	0.34	40.08	72.16	113.26
**2a**	0.60	0.39	0.47	1.38	19.36	45.00
**3**	0.67	0.66	0.42	62.39	104.70	137.52
**3a**	0.51	0.51	0.43	41.11	75.05	94.90
**4**	0.59	0.49	0.24	49.88	50.11	52.88
**4a**	0.61	0.52	0.43	8.28	46.73	71.83

**Table 3:** Peak temperatures and denaturation heat obtained for collagen at pH 3.5 and pH 7.5.

Sample	T_peak1_, °C	T_peak2_, °C	T_peak3_, °C	Denaturation Heat, J/g
**1** (pH 3.5)	36.10	41.06	45.82	0.27
**1** (pH 7.5)	36.74	43.93	47.15	0.29

**Table 4:** Peak temperatures and denaturation heat of collagen in different systems at pH 7.5.

Sample	T_peak1_, °C	T_peak2_, °C	T_peak3_, °C	T_peak4_, °C	Denaturation Heat, J/g
**1**	36.74	43.93	47.15	-	0.23
**1a**	37.22	44.15	47.42	-	0.27
**2**	37.60	43.89	50.32	-	0.29
**2a**	42.44	43.88	54.24	57.33	0.24
**3**	32.96	40.31	43.71	50.95	0.17
**3a**	40.80	43.70	50.63	54.04	0.13
**4**	37.46	43.93	51.43	54.15	0.27
**4a**	38.73	44.04	50.43	-	0.26

## Data Availability

Not applicable.

## Supplementary Materials

The following are available online. Figure S1: Rheograms for UVA-irradiated collagen (sample **1a**) at the indicated temperatures (collagen 0.25%, pH 7.5), Figure S2: Rheograms for the collagen-riboflavin system (sample **2**) at the indicated temperatures (collagen 0.25%, riboflavin 0.1%, pH 7.5), Figure S3: Rheograms for the UVA-irradiated collagen-riboflavin system (sample **2a**) at the indicated temperatures (collagen 0.25%, riboflavin 0.1%, pH 7.5), Figure S4: Rheograms for the collagen-hyaluronic acid system (sample **3**) at the indicated temperatures (collagen 0.25%, hyaluronic acid 0.1%, pH 7.5), Figure S5: Rheograms for the UVA-irradiated collagen-hyaluronic acid system (sample **3a**) at the indicated temperatures (collagen 0.25%, hyaluronic acid 0.1%, pH 7.5), Figure S6: Rheograms for the collagen-riboflavin-hyaluronic acid system (sample **4**) at the indicated temperatures (collagen 0.25%, riboflavin 0.1%, hyaluronic acid 0.1%, pH 7.5), Figure S7: Rheograms for the UVA-irradiated collagen-riboflavin-hyaluronic acid system (sample **4a**) at the indicated temperatures (collagen 0.25%, riboflavin 0.1%, hyaluronic acid 0.1%, pH 7.5), Figure S8: PeakFit decomposition of DSC thermograms for collagen at (A) pH 3.5 and (B) pH 7.5, Figure S9: Molecular structures and spin trapping mechanisms of the spin traps used, Figure S10: The spectrum of riboflavin/DMPO solution in the absence (black) and in the presence (cyan) of UVA light. Collagen extraction and purification procedure [60,61]. Table S1: Contributions of DMPO spin adducts detected in solutions of tear proteins, in the presence of riboflavin, after UVA exposure.

## Sample Availability

Samples are available from the authors.
