# Supplementary material for: Model Systems for Evidencing the Mediator Role of Riboflavin in the UVA Cross-Linking Treatment of Keratoconus
Source: Molecules. 2021 Dec 29;27(1):190. doi: 10.3390/molecules27010190 (PMC8746477; doi:10.3390/molecules27010190)
Supplement: Supplementary file 1 [file molecules-27-00190-s001.zip › molecules-1482540-supplementary.pdf]

Model systems for evidencing the mediator role of riboflavin in the UVA cross-linking treatment of keratoconus

Mihaela Monica Constantin[1], Cătălina Gabriela Corbu[1,2], Sorin Mocanu[3], Elena Irina Popescu[3], Marin Micutz[4*], Teodora Staicu[4], Raluca Şomoghi[5,6], Bogdan Trică[6], Vlad Tudor Popa[3], Aurica Precupas[3*], Iulia Matei[3] and Gabriela Ionita[3*]

[1] Oftaclinic Clinic, Bd. Marasesti 2B, 040254 Bucharest, Romania
[2] Clinical Hospital of Ophthalmologic Emergencies, Alexandru Lahovari 1 Square, 010464 Bucharest, Romania
[3] "Ilie Murgulescu" Institute of Physical Chemistry of the Romanian Academy, Splaiul Independentei 202, Bucharest 060021, Romania
[4] Department of Physical Chemistry, Faculty of Chemistry, University of Bucharest, Bd. Regina Elisabeta 4-12, Bucharest 030018, Romania
[5] Chemistry Department, Faculty of Petroleum Technology and Petrochemistry, Petroleum-Gas University of Ploiesti, Bd. Bucuresti 39, Ploiesti 100680, Romania
[6] Department of Bioresources, National Institute for Research & Development in Chemistry and Petrochemistry—ICECHIM, Splaiul Independentei nr. 202, Sector 6, 060021 Bucharest, Romania

* Correspondence: ige@icf.ro (GI); micutz@gw-chimie.math.unibuc.ro (MM); aprecupas@icf.ro (AP)

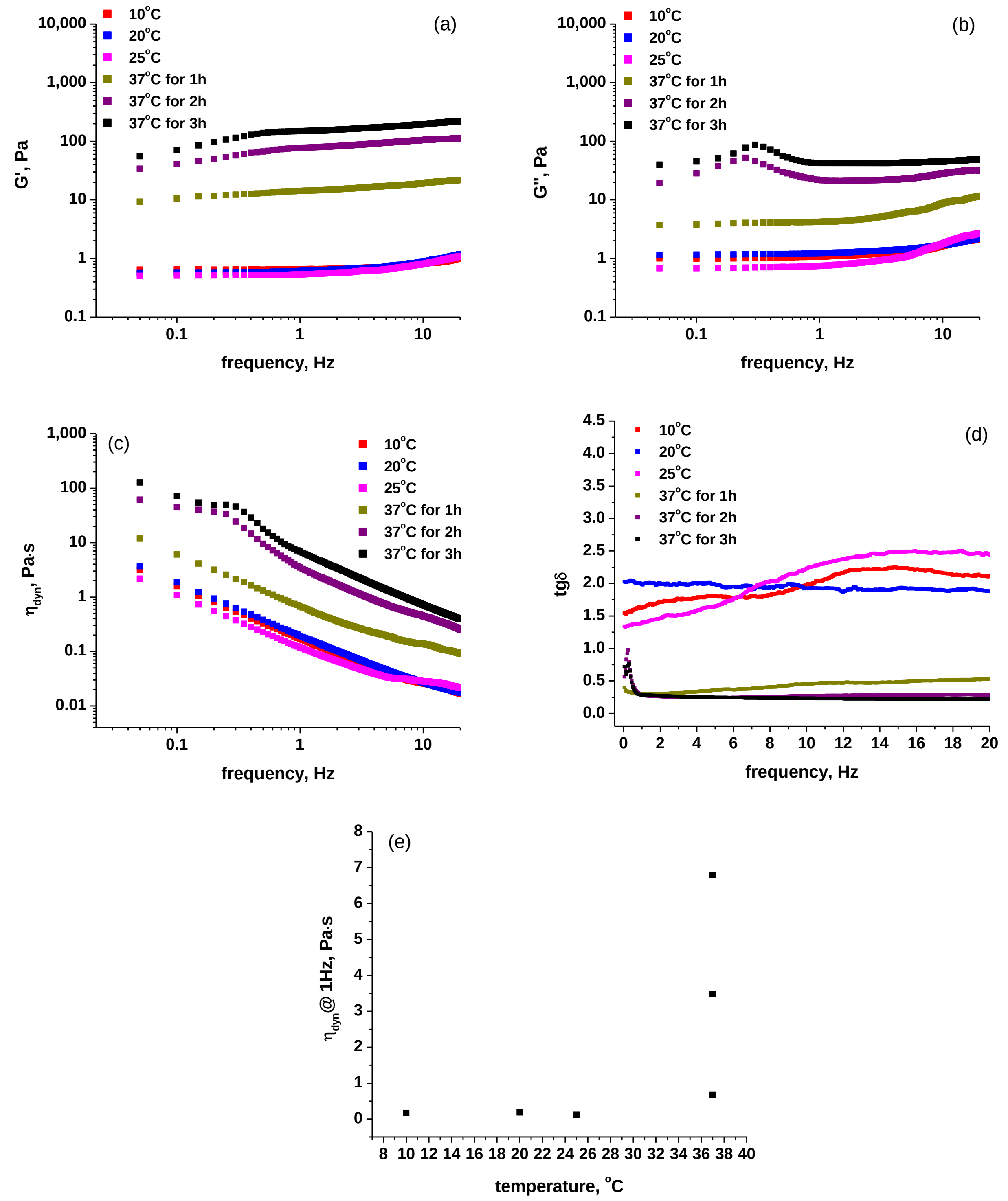

Figure S1. Rheograms for UVA-irradiated collagen (sample 1a) at the indicated temperatures (collagen 0.25%, pH 7.5).

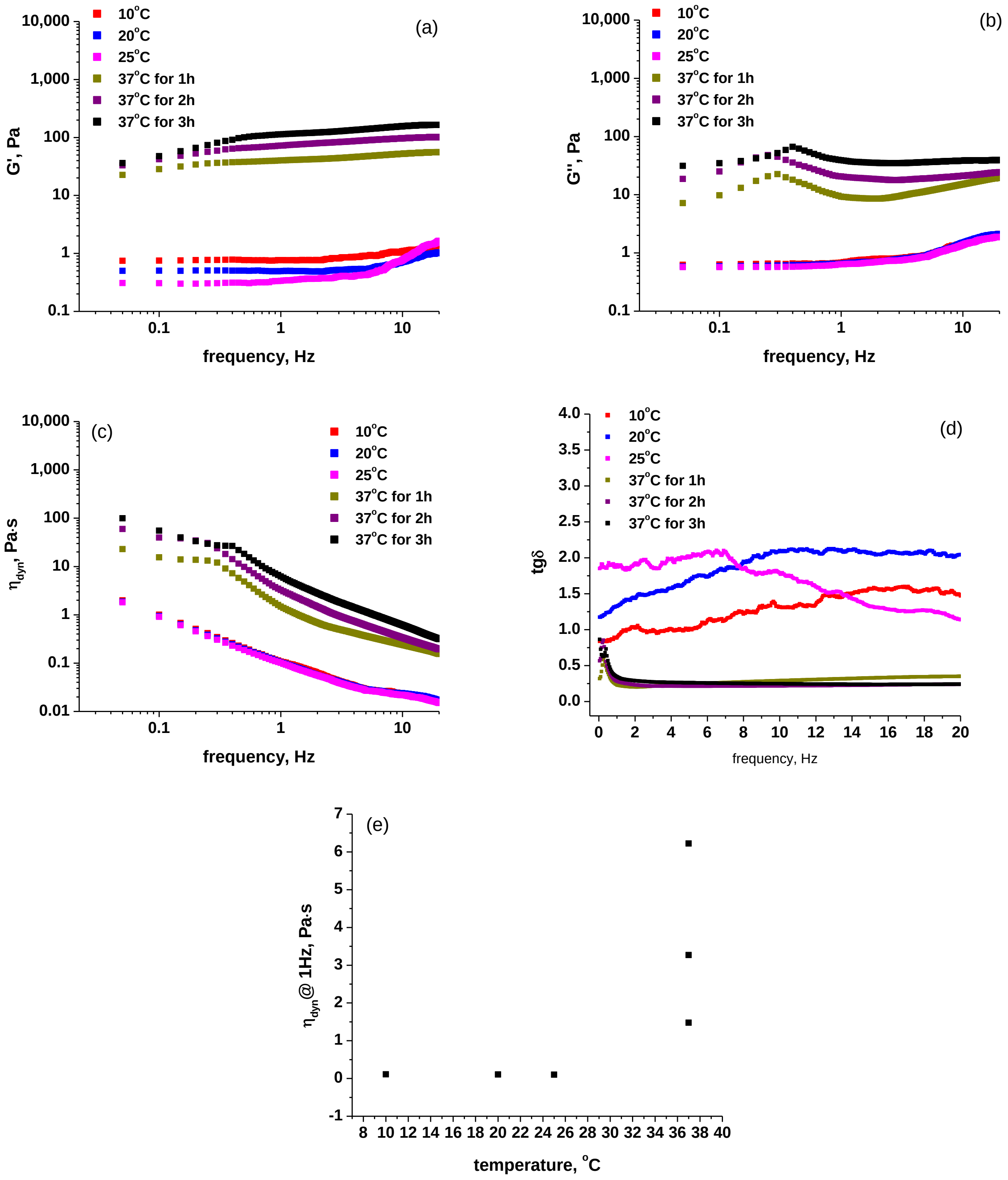

Figure S2. Rheograms for the collagen-riboflavin system (sample 2) at the indicated temperatures (collagen 0.25%, riboflavin 0.1%, pH 7.5).

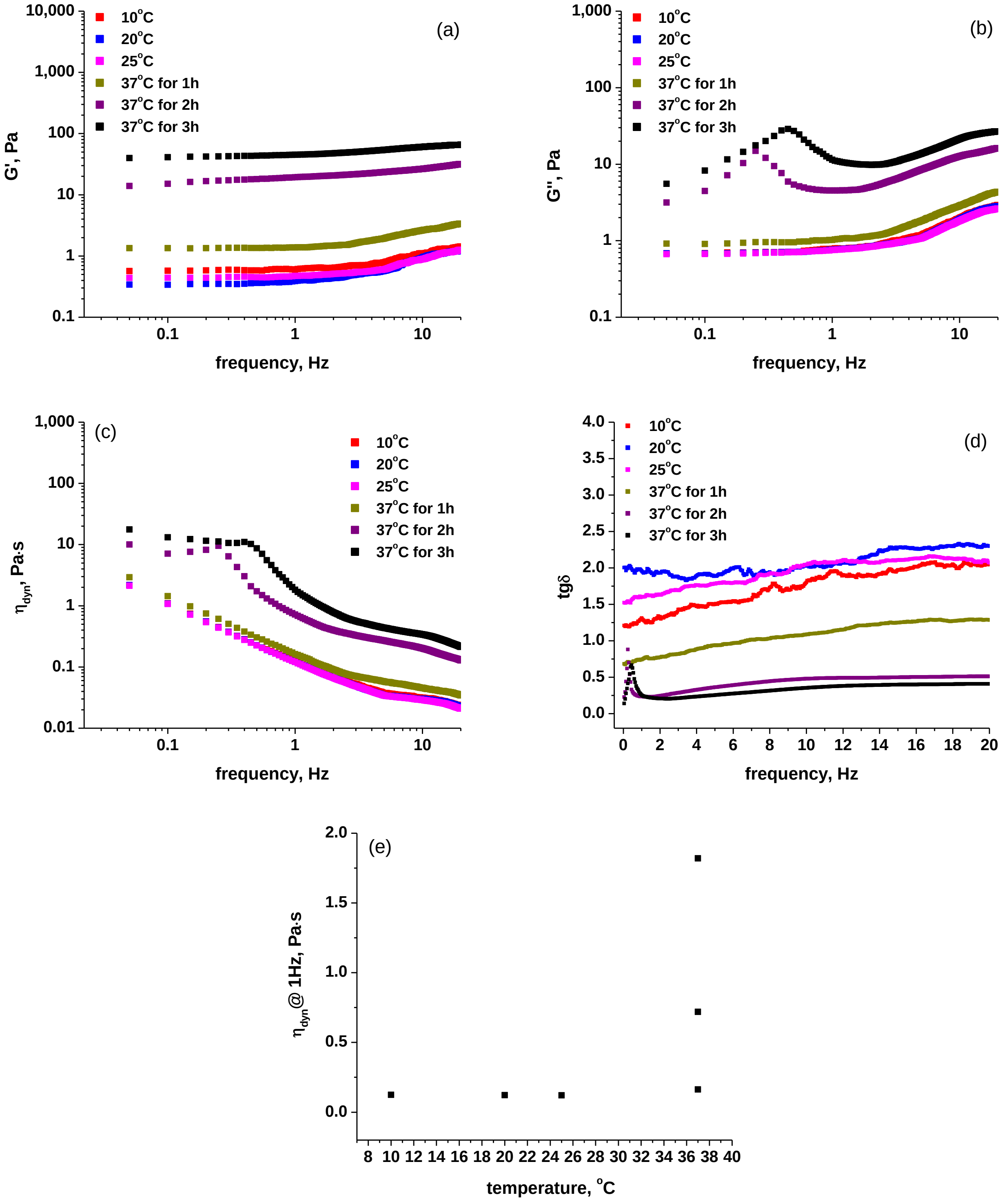

Figure S3. Rheograms for the UVA-irradiated collagen-riboflavin system (sample 2a) at the indicated temperatures (collagen 0.25%, riboflavin 0.1%, pH 7.5).

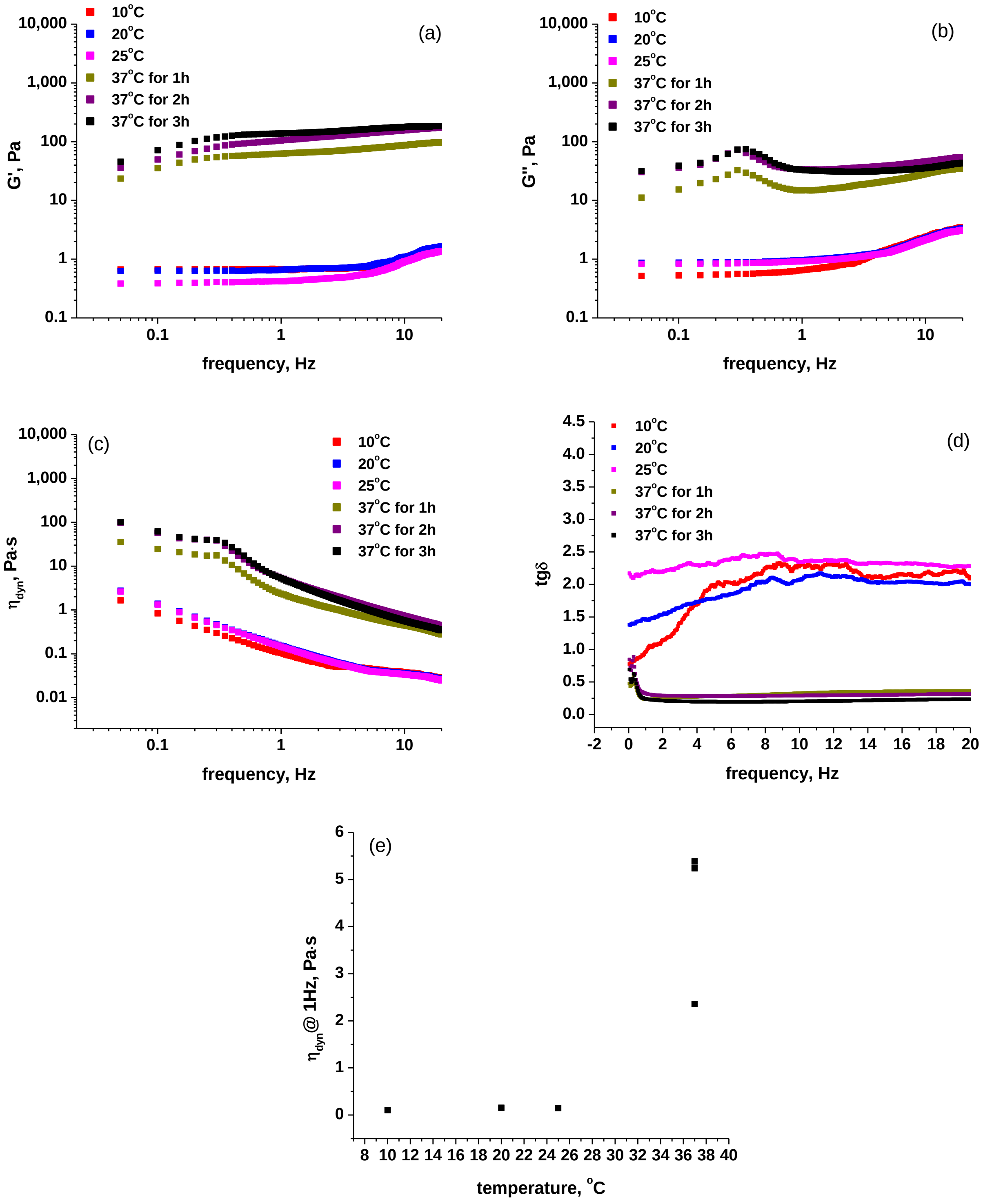

Figure S4. Rheograms for the collagen-hyaluronic acid system (sample 3) at the indicated temperatures (collagen 0.25%, hyaluronic acid 0.1%, pH 7.5).

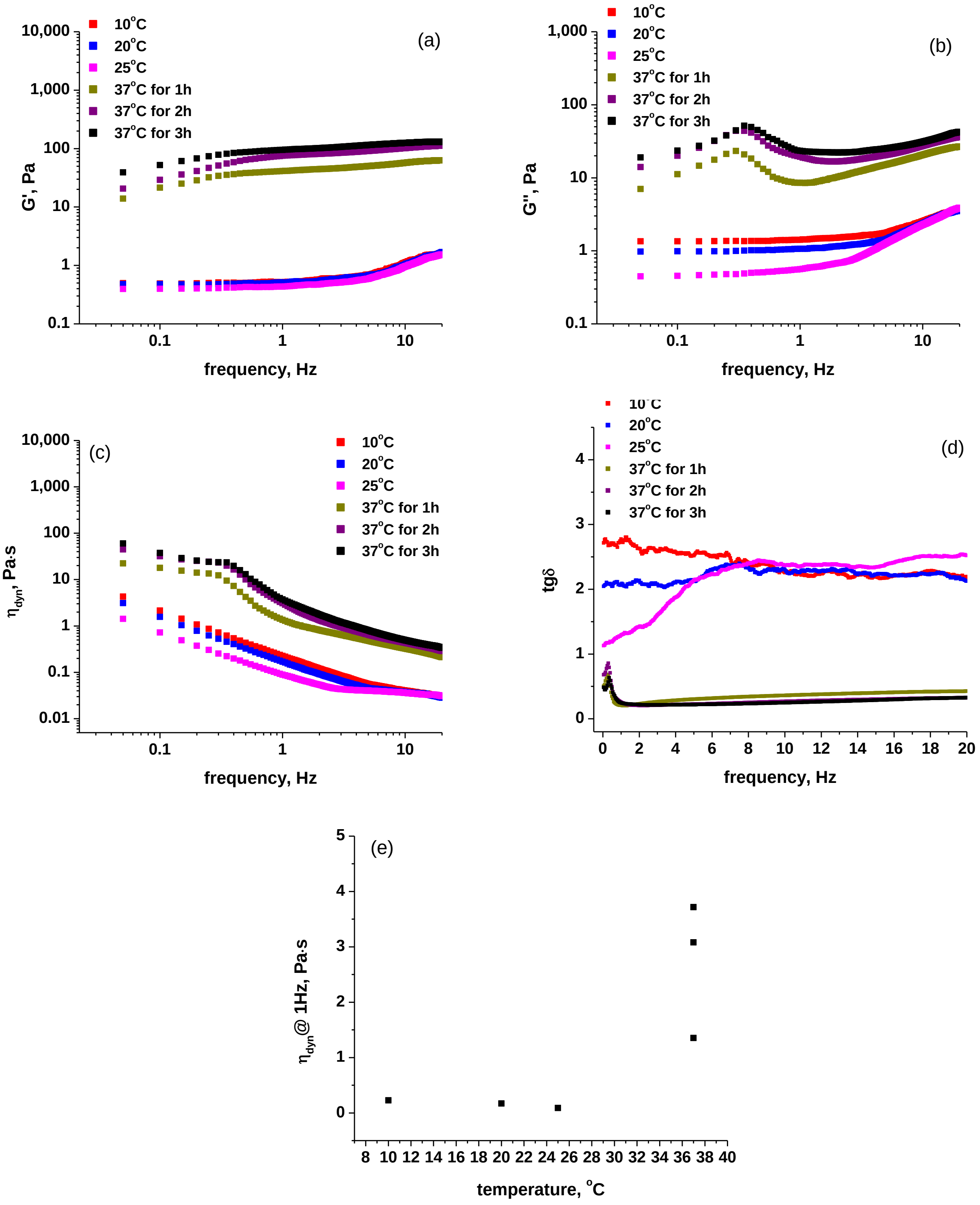

Figure S5. Rheograms for the UVA-irradiated collagen-hyaluronic acid system (sample 3a) at the indicated temperatures (collagen 0.25%, hyaluronic acid 0.1%, pH 7.5).

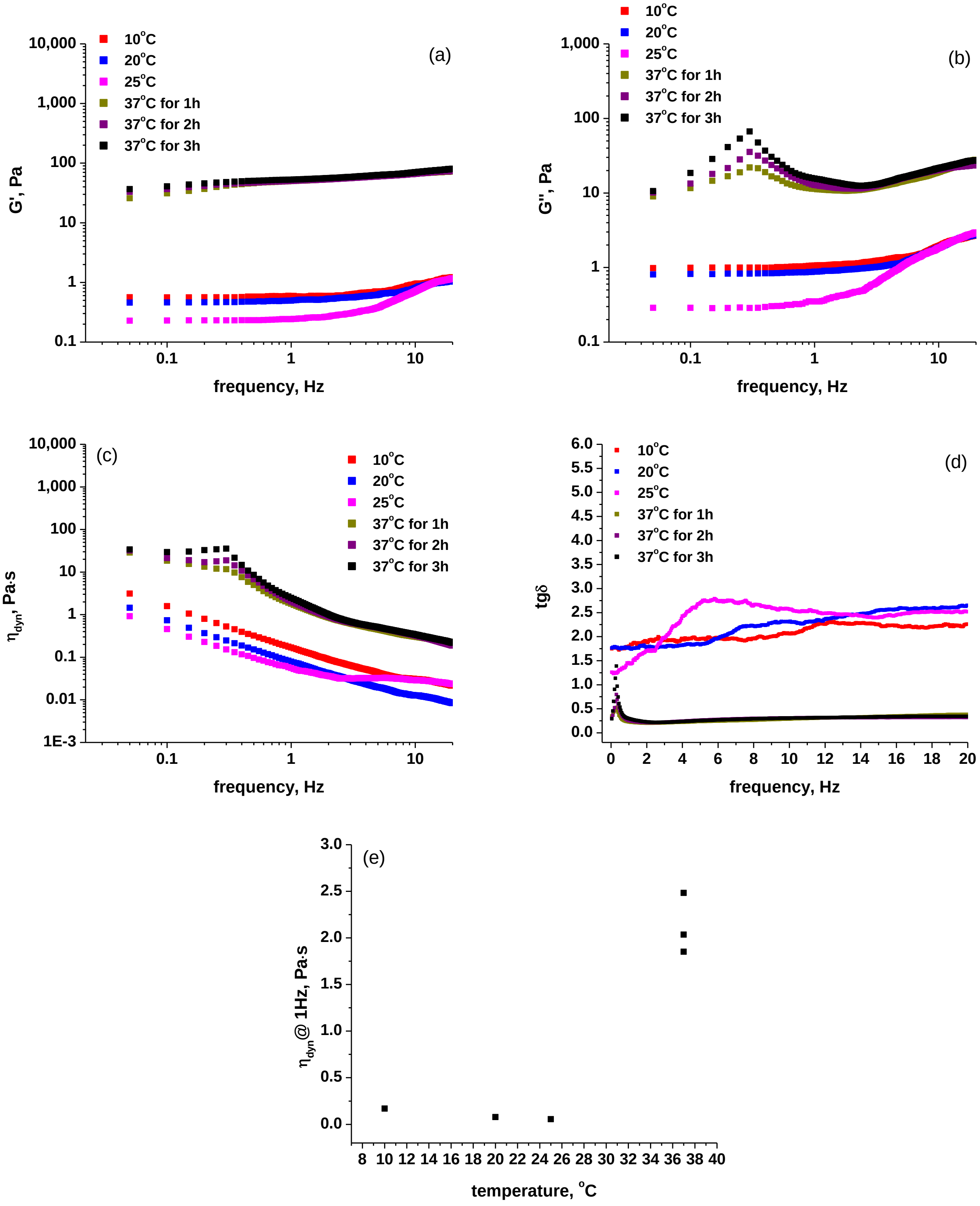

Figure S6. Rheograms for the collagen-riboflavin-hyaluronic acid system (sample 4) at the indicated temperatures (collagen 0.25%, riboflavin 0.1%, hyaluronic acid 0.1%, pH 7.5).

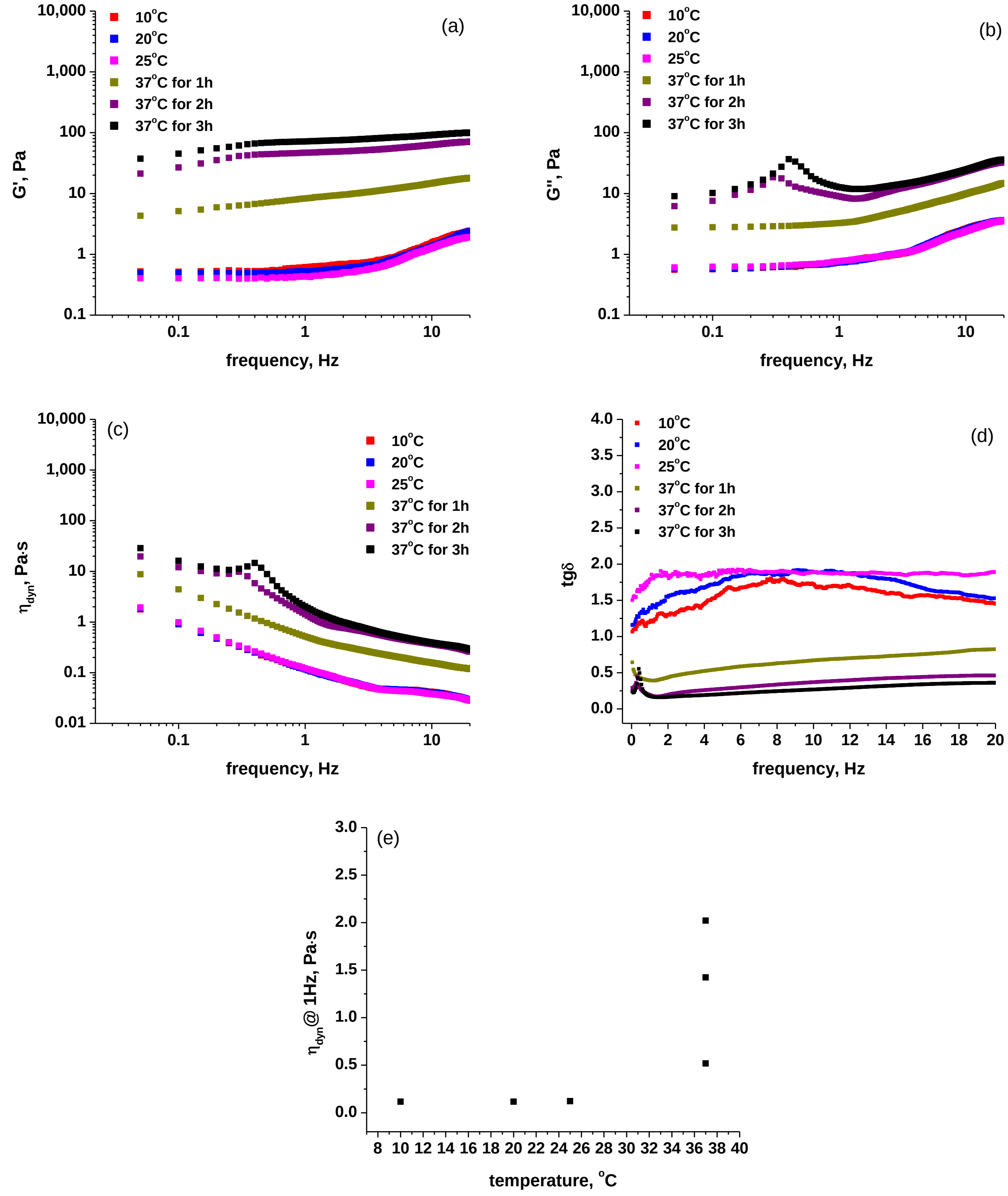

Figure S7. Rheograms for the UVA-irradiated collagen-riboflavin-hyaluronic acid system (sample 4a) at the indicated temperatures (collagen 0.25%, riboflavin 0.1%, hyaluronic acid 0.1%, pH 7.5).

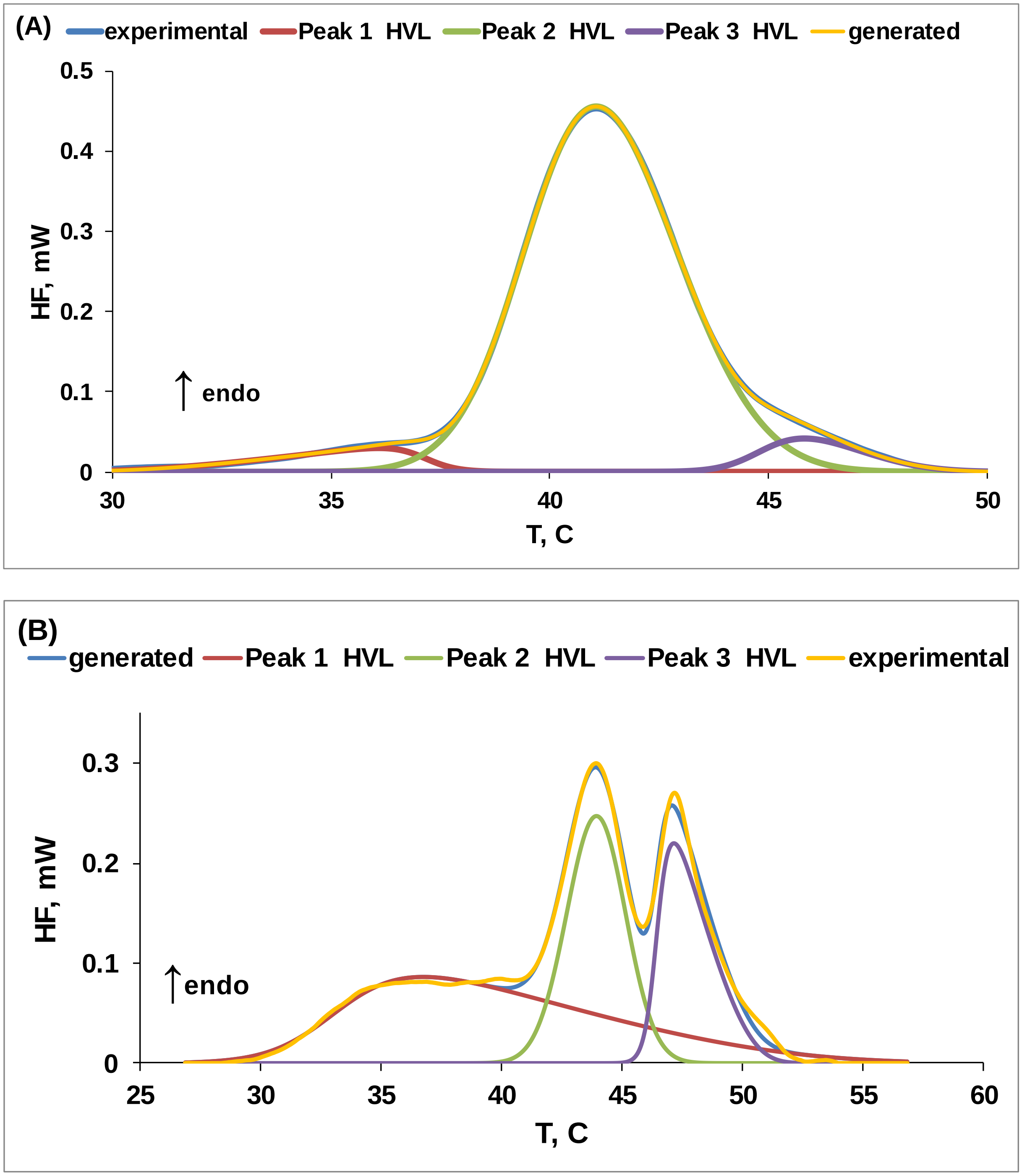

Figure S8. PeakFit decomposition of DSC scans obtained for collagen thermal denaturation at (A) pH 3.5 and (B) pH 7.5.

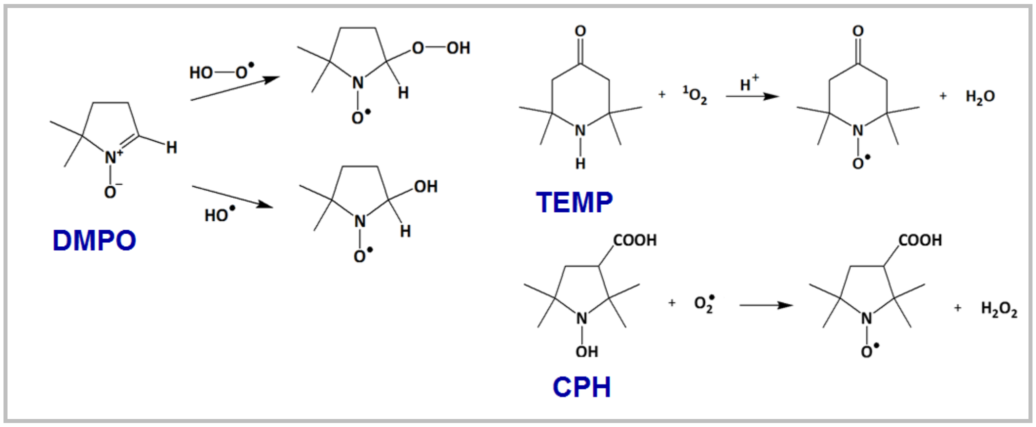

Figure S9. Molecular structures and spin trapping mechanisms of the spin traps used.

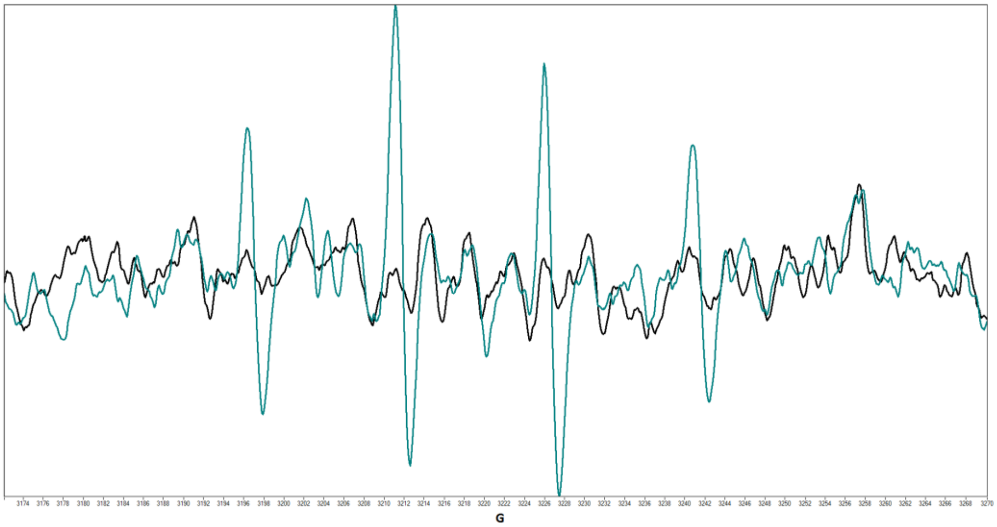

Figure S10. The spectrum of riboflavin/DMPO solution in the absence (black) and in the presence (cyan) of UVA light.

Table S1. Contributions of DMPO spin adducts detected in solutions of tear proteins, in the presence of riboflavin, after UVA exposure

	Spin adduct		
**System**	**$^{\bullet}$DMPO-OH**	**$^{\bullet}$DMPO-C**	**DMPO-NO$^{\bullet}$**
HSA	28	67	5
LF	26	71	3
LYZ	20	79	1
HSA+LF+LYZ	16	81	3
HSA+LF+LYZ+HA*	68	24	8

* HA – hyaluronic acid

Collagen extraction and purification

Collagen type I was extracted from calfskin, as it is known that the extracellular matrix of skin is a very useful collagen type I-rich source [18,60,61]. We employed a modified procedure adapted from experimental data reported elsewhere [references 1-8 in the main article]. Several steps were carried out starting from an initial amount of calfskin: mechanical and chemical unhairing, noncollagenous proteins and fats removal, extraction and purification of collagen type I.

About 100 g of calf skin were unhaired mechanically by shaving, then chemically in 200 mL aqueous limed liquor (containing 2 g of Na_2S and 6 g of CaO) for two days. The unhaired skin was then delimed for 30 min by adding 3 g of NH_4Cl and 5 mL of concentrated HCl (37–38%) under stirring, followed by extensive rinsing with tap/distilled water. The pretreated calfskin was cut into tiny pieces and minced together with ice flakes.

Noncollagenous proteins removal from minced calfskin was performed in 1000% (with respect to calfskin weight) aqueous solution of 0.1 M NaOH for two days, under stirring at 4-8°C. The deproteinized skin was then abundantly rinsed with distilled water at room temperature. To extract the fats, the calfskin was immersed in aqueous solution (10%) of n-butanol (skin/n-butanol solution = 1/20 by weight) and maintained at 4-8°C under stirring for 24 h, with replacing the butanol solution every 6 h. Rinsing the deproteinized-defatted skin with distilled water until the butanol odor vanished was carried out at room temperature.

The most important phase of extraction and purification consists in collagen extraction with 20 volume of aqueous solution of 0.5% acetic acid containing 1% pepsin with respect to calfskin weight at 4-8°C for three days. The supernatant of the extracted solution was separated by filtrating using a two-layer cheesecloth and then by centrifugation (18000×g) for 15 min. To inactivate pepsin, the supernatant mainly containing collagen type I was dialyzed (molecular weight cut-off 12 kDa) against a solution of 0.02 M disodium phosphate (pH 7.4–7.5) at 4–8°C. During dialysis (24 h under moderate stirring), the disodium phosphate solution was changed four times and, finally, the precipitated collagen was collected via centrifugation (18000×g, 15 min, 4–8°C). The collagen (consisting of collagen type I as main component mixed with collagen type III as minor component) was dissolved in aqueous solution of 0.5 M acetic acid to an estimated concentration of 0.5 g/dL. After a salting out step (dialysis against aqueous solution of 1M NaCl in 0.5 M acetic acid, 24 h under stirring, 4-8°C, changing dialyzing solution every 6 h) and centrifugation under the same conditions, the collagen was redissolved in 0.5 M acetic acid (4–8°C, to an estimated concentration of ca. 0.5 g/dL). To separate collagen type III, the collagen type I-III solution in 0.5 M acetic acid was salted out in 1.5 M NaCl (in 0.05 M Tris solution of pH 7.4) via dialysis (two days, moderate stirring, 4–8°C, dialysis solution changed every 6 h) and, after centrifugation (keeping the same operational parameters), the supernatant without collagen type III was salted out in 2.5 M NaCl (in 0.05 M Tris of pH 7.4) through dialysis (under the above-mentioned conditions). Collagen type I so precipitated was collected by centrifugation (as described above) and finally redissolved in an aqueous solution of 0.1 M acetic solution to finally give an acetic solution of collagen type I of about 0.5 % ready to be used. The high purity of collagen type I so obtained as atelocollagen type I was assessed by sodium dodecyl sulfate-polyacrylamide gel electrophoresis (SDS-PAGE), as can be seen in Figure S11.

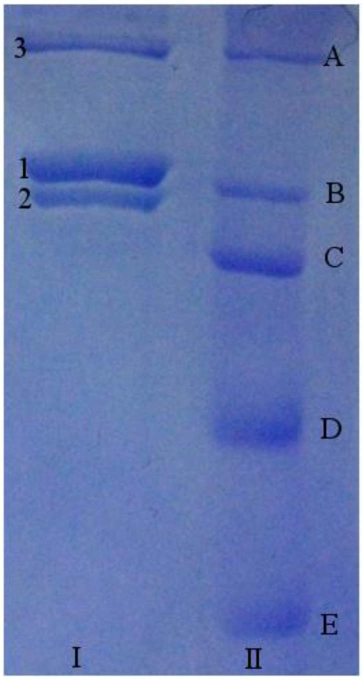

Figure S11. Electropherogram of atelocollagen type I (lane I) by comparison with that of calibration references (A - myosin, rabbit muscle – 200 kDa, B - β-galactosidase, *E. coli* – 116 kDa, C - phosphorilase b, rabbit muscle – 97 kDa, D - bovine serum albumin – 66 kDa, E - glutamic dehydrogenase, bovine liver – 55 kDa, SigmaMarker, Sigma; lane II). The purity of protein is revealed by the migrated bands associated with α1(I) and α2(I) polypeptide chains and β fraction of collagen type I (1, 2 and 3, respectively, in lane I).
